# Mitochondrial Oxidative Stress and “Mito-Inflammation”: Actors in the Diseases

**DOI:** 10.3390/biomedicines9020216

**Published:** 2021-02-20

**Authors:** Simone Patergnani, Esmaa Bouhamida, Sara Leo, Paolo Pinton, Alessandro Rimessi

**Affiliations:** 1 Department of Medical Sciences and Laboratory for Technologies of Advanced Therapies (LTTA), University of Ferrara, 44121 Ferrara, Italy; simone.patergnani@unife.it (S.P.); bhmsme@unife.it (E.B.); sara.leo@unife.it (S.L.); paolo.pinton@unife.it (P.P.); 2 Center of Research for Innovative Therapies in Cystic Fibrosis, University of Ferrara, 44121 Ferrara, Italy

**Keywords:** oxidative stress, mitochondria, mito-inflammation, cancer, pulmonary diseases, gastrointestinal disorders, neurodegenerative disorders, diabetes

## Abstract

A decline in mitochondrial redox homeostasis has been associated with the development of a wide range of inflammatory-related diseases. Continue discoveries demonstrate that mitochondria are pivotal elements to trigger inflammation and stimulate innate immune signaling cascades to intensify the inflammatory response at front of different stimuli. Here, we review the evidence that an exacerbation in the levels of mitochondrial-derived reactive oxygen species (ROS) contribute to mito-inflammation, a new concept that identifies the compartmentalization of the inflammatory process, in which the mitochondrion acts as central regulator, checkpoint, and arbitrator. In particular, we discuss how ROS contribute to specific aspects of mito-inflammation in different inflammatory-related diseases, such as neurodegenerative disorders, cancer, pulmonary diseases, diabetes, and cardiovascular diseases. Taken together, these observations indicate that mitochondrial ROS influence and regulate a number of key aspects of mito-inflammation and that strategies directed to reduce or neutralize mitochondrial ROS levels might have broad beneficial effects on inflammatory-related diseases.

## 1. Introduction

Free radicals and other reactive oxygen species (ROS) are frequently associated with being harmful. However, several physiological functions (differentiation, cellular signaling, phosphorylation/dephosphorylation events and apoptosis) are dependent on the presence of reactive species inside the cells [1]. To regulate these cellular functions, the ROS production must be kept low. When it increases, ROS become dangerous, determining undesirable effects on cellular structures, including intracellular organelles, and participating in the onset and/or progression of several human disorders, such as neurodegenerative disorders, cancer, pulmonary, and cardiovascular diseases [2,3,4,5]. ROS can be produced at cytosolic levels by the NADPH oxidase (NOX) and the nitric oxide (NO) synthase enzyme. The well-characterized source of cytosolic ROS is NOX2, which produces anion superoxide (O_2_·^−^) through NAPDH electron exchange [6]. Alternatively, significant amounts of ROS are generated in the peroxisomes compartment by oxidases and NO synthases that produce hydrogen peroxide (H_2_O_2_) and NO, respectively [7]. The endoplasmic reticulum (ER) represents a place where high amounts of ROS are produced. In this compartment, ROS result in being the byproduct of oxidases and oxygenase involved during the protein folding process, such as ER oxidoreductin 1 [8]. Moreover, ER present iron deposits that contribute to form the reactive species hydroxyl radical (·OH), but undoubtedly, mitochondria represent the primary source of ROS (mitochondrial ROS, mtROS) for the cell [9].

Mitochondria are essential semi-autonomous cellular organelles with a double membrane composed by an inner (IMM) and an outer membrane (OMM). OMM separates the mitochondria from cytoplasm and it may be freely traversed by many proteins (5000 daltons or less), ions, such as Calcium (Ca^2+^), and metabolites, while the larger molecules are imported by specific translocase [10,11,12]. IMM is highly specialized and permits the passage of small molecules, including oxygen, carbon dioxide, and water. The passage throughout IMM of any other macromolecules is permitted by the action of dedicated translocase, like TIM (translocase of the inner membrane). Within the organelle, OMM and IMM allow the formation of two different compartments: the intermembrane space (IMS) and the matrix [13]. IMS plays a crucial role in regulating different cellular activities, such as mitochondrial respiration, proteins transport, lipid homeostasis and metal ion exchange; while the matrix contains the mitochondrial DNA (mtDNA), mitochondrial ribosomes and multiple diverse metabolic pathways [tricarboxylic acid (TCA) cycle, β-oxidation, and heme synthesis], that modulate the assembly and genes expression, as well as, Ca^2+^ uptake which is critically important to cellular function [14,15,16] mtDNA is a closed circle double-stranded DNA, without histones and of effective repair mechanism. mtDNA encodes for 37 genes, including 13 components of the mitochondrial electron transport chain (mETC) [17]. Mitochondrial matrix is also the site of a series of enzyme that convert pyruvate and fatty acid in acetyl-CoA. Once produced, acetyl-CoA enters in TCA cycle which produces NADH and FADH2 that are used by mETC [18]. 

**Figure 1:**
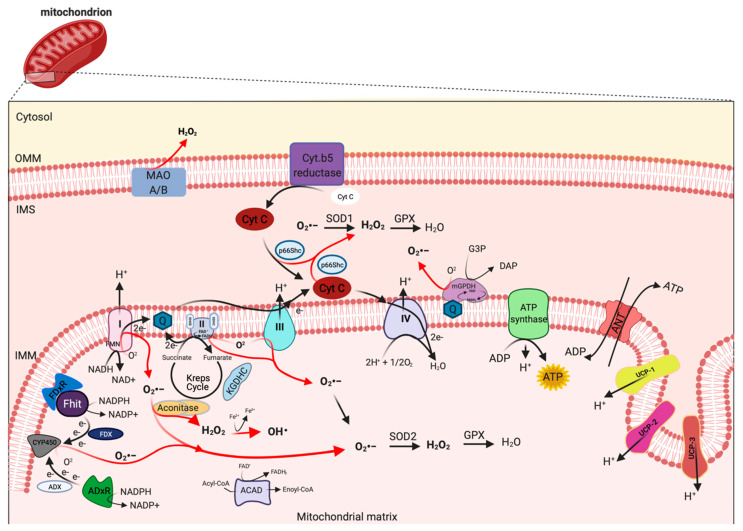
Mitochondrial sites of ROS production. Mitochondrial complex I and III of respiratory chain are the principal sites of O_2_^•−^ production within a cell, which can be converted to H_2_O_2_ by superoxide dismutase (SOD1 and SOD2) enzymes. H_2_O_2_ in turn is rapid neutralized to H_2_O and oxygen by glutathione peroxidase (GPX). However, other mitochondrial proteins, localized from OMM to matrix, may also contribute to mtROS production, including monoamine oxidase A and B (MAO A/B), cytochrome (Cyt.) b5 reductase, mitochondrial glycerol-phosphate dehydrogenase (mGPDH), p66Shc, Fhit with ferredoxin reductase (FDxR), adrenodoxin reductase (ADxR)-adrenodoxin (ADX)-cytochrome P450scc (CYP450) system, α-ketoglutharate dehydrogenases (KGDHC), acyl-CoA dehydrogenases (ACAD) and aconitase. This figure has been created with “BioRender.com.”.

**Figure 2:**
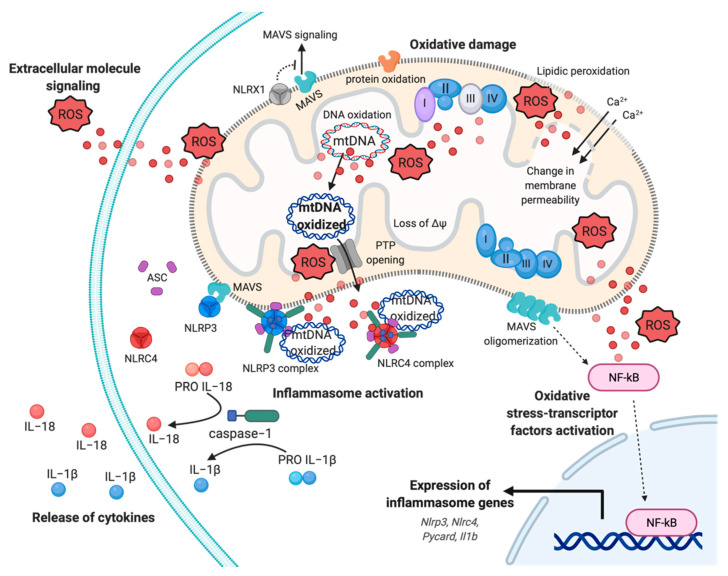
Mitochondrial ROS-induced inflammatory response. Representation of multifaceted aspects of mitochondrial ROS in inflammation. Increased mtROS cause oxidative damage to mitochondrial membrane, with events of lipidic peroxidation and changes in membrane permeability, molecules, proteins, and mtDNA, which contribute to mitochondrial dysfunction and exacerbation of mtROS production. The dissemination of mtROS actives the redox-sensitive transcription factor NF-kB, inducing the expression of inflammasome genes, such as Nlrp3, Nlrc4, and Il1b genes. In turn, the mtROS and mtDNA promote the cytokines release mediating the inflammasome NLRP3 and NLRC4 complex activation, through the recruitment of pro-caspase−1 and the processing of pro-IL−1 and pro-IL−18. Finally, mtROS are reversed to extracellular milieu to sustain and exacerbate the inflammatory responses, affecting proximal cells and conditioning the inflammatory microenvironment. Abbreviations: Reactive oxygen species, ROS; Interleukin−18, IL−18; Interleukin−1, IL−1; NLR Family CARD Domain Containing 4, NLRC4; ASC; NLR Family Pyrin Domain Containing 3, NLRP3; Mitochondrial antiviral-signaling protein, MAVS; mitochondrial deoxyribonucleic acid, mtDNA; NLR Family member X1 precursor, NLRX1; Calcium, Ca^2+^; Nuclear factor kappa-light-chain-enhancer of activated B cells, NF-kB; Mitochondrial membrane potential, ΔΨ. This figure has been created with “BioRender.com.”.

mETC is composed by four complexes (I-IV) that are embedded in the IMM and are responsible to create the electrochemical gradient required to generate ATP. Notably, complex-I (NADH-CoQ reductase) and complex III (cytochrome c reductase) represent the primary sites of production of mitochondrial O_2_·^−^ (Figure 1). This byproduct of the mitochondrial respiration is also considered the most quantitatively important mtROS source in higher organisms [2]. However, mitochondria are not only an ROS-producing compartment but, in turn, are their targets. In particular, mitochondrial phospholipids and DNA are susceptible to mtROS-induced oxidative damage (Figure 2). Mitochondrial phospholipids loss their permeability and fluidity compromising the functioning of all factor associated to the membrane, in particular mETC members and ion channels. mtDNA loses its integrity, acquiring mutations and reducing in the number of mtDNA copies. In turn, mtDNA mutations can determine further alteration in mitochondrial functioning and signaling that may be determinant for various human diseases [19]. To avoid accumulating oxidative damages, mitochondria have evolved different mechanisms to reduce the mtROS-induced oxidative damage or eliminate injured mitochondria. The mitochondrial antioxidant systems and the mitochondrial stress responses (including mitophagy, mitochondrial unfolded protein response (mtUPR), and apoptosis) represent some classical examples [2]. In addition, it is widely accepted that alterations in mitochondrial functions (like in Ca^2+^ dynamics and/or in lipid transfer from ER to mitochondria) modulate the mtROS production [2]. Thus, increased levels of mitochondrial Ca^2+^ activate ROS-generating enzyme and the formation of radicals.

Clearly, mitochondria and mtROS production are primary signal hub for the cells. It is not surprising that diverse studies highlight their involvement in pathogenesis of various diseases, including neurodegenerative disorders, cancer, viral and bacterial infection, cardiovascular diseases, metabolic syndromes, and autoimmune disorders [20]. In particular, it has been proposed that mitochondrial dysfunction and excessive mtROS levels sustain inflammation in these pathological conditions. In these contexts, mtROS-induced inflammation acts as a feedback system creating a stressful environment, where the exacerbation of inflammation provokes tissue damage and becomes a chronic event [20]. Considering the importance of oxidative stress in inflammation, it is easy to speculate that therapeutic manipulations aimed to prevent oxidative damage, targeting the mitochondrial (dys)function and generating an antioxidant power, may represent an opportunity to disrupt the reciprocal relation between mtROS and “mito-inflammation”. A new concept that identifies the compartmentalization of the inflammatory process in which the mitochondrion acts as central regulator, checkpoint, and arbitrator.

The present review will summarize the current literature surrounding the role of mitochondria in ROS production and signaling in both physiology and pathophysiology. In particular, it will be discussed how mitochondrial oxidative stress regulates diverse human diseases throughout the modulation of “mito-inflammation”, with a focus on the therapeutic mitochondria-targeted strategies for avoid mtROS production and reduce the oxidative stress, in order to counteract, or better, prevent the inflammatory state at the base of pathological conditions.

## 2. Mitochondria: A ROS Production Machinery

Mitochondria are widely recognized as a source of ROS production within most mammalian cells in both physiological and pathological conditions. The generation of mtROS from mitochondria was first discovered during the early 1970s [21,22]. According to estimates, 1–2% of total cellular oxygen consumption is going to ROS production [23].

mtROS are produced as byproduct of bioenergetic metabolism during the process of oxidative phosphorylation (OXPHOS) and formed by one-electron transfers, generating O_2_^•−^ that can be converted to H_2_O_2_ by superoxide dismutase (SOD) enzymes [24]. Multiple sites of mtROS production have long been identified in the organelle and mtROS can take place both in the mitochondrial matrix by the core metabolic machinery present in the IMM and in the intermembrane space [25]. Generation of mtROS mainly takes place at the ETC, located on the IMM, during the process of OXPHOS [26], which includes the major sites of the respiratory chain Complex I (NADH dehydrogenase (ubiquinone), 45 protein subunits), Complex III (ubiquinol-cytochrome c reductase, 10 proteins subunits), and also the dihydrolipoamide dehydrogenase enzyme [27,28]. Complex I generates O_2_^•−^ by reducing flavin mononucleotide (FMN) site on complex I and reversing electron transfer from the coenzyme Q (CoQ) pool back to complex I [2]. Basically, complex II (succinate dehydrogenase) was not considered a source of ROS per se, instead its contribution to ROS generation is linked to reverse electron transfer, in which electrons are transferred from succinate to ubiquinone through complex II and then back to complex I [29]. Complex II consists of four subunits. Two subunits are located on the matrix side of the IMM: SDHA (succinate dehydrogenase), the flavoprotein subunit covalently bound a FAD cofactor, which removes electrons from succinate; SDHB, the iron-sulfur protein subunit, contains the binding site of the substrate succinate, three clusters (2Fe-2S), (4Fe-4S), and (3Fe-4S) that mediate electron transfer to the ubiquinone molecule; and SDHC and SDHD the two anchor subunits to the IMM, the assembly factors participate in the biogenesis of complex II [30,31,32]. Brand’s group demonstrated that complex II can produce superoxide through flavin adenine nucleotide (FAD) [33]. Indeed, it has been suggested that other less well-described sites may also participates in ROS production including, the electron transferring flavoprotein/ETF:Q oxidoreductase (ETF/ETF:QOR) system of fatty acid β-oxidation [34], dihydroorotate dehydrogenase [35], and proline dehydrogenase [36]. These different mtROS’s sites have distinct signaling roles and subsequently the primary production sites change under different physiological conditions [37]. Indeed, the production of radicals from the mitochondrial respiratory chain is conditioned by multiple factors, including mitochondrial membrane potential, metabolic state of mitochondria and oxygen levels [38].

Other mitochondrial proteins may participate to increment the mtROS production. In particular, the mitochondrial enzyme dehydrogenase of α phosphate dehydrogenase (glycerol α phosphate dehydrogenase, mGPDH), located at the outer surface of the IMM, is a flavoprotein containing FAD and serves as an electron shuttle linking the cytosolic NADH/ NAD recycling to the mETC implicated in lipid metabolism capable of producing cytosolic NAD^+^ from the NADH formed in glycolysis [39]. However, it is unequally expressed in numerous mouse tissues mediating the generation of H_2_O_2_ [40]. Therefore, other potential sources of mtROS are still more poorly explored, such as acyl-CoA dehydrogenases (ACAD), which are flavoproteins and have increasingly been recognized as oxidant sources in mitochondria, involved in lipid catabolism [41]. Both of these proteins are implicated in mtROS production in tissues during the oxidation lipid-derived substrates [42]. The mitochondrial enzyme adrenodoxin reductase (ADxR)-adrenodoxin (ADX)-cytochrome P450scc (CYP450) system (cholesterol side chain cleavage) is also involved in superoxide generation and is coupled with NADPH in the mitochondrial matrix [43]. The onco-suppressor Fhit protein, imported into mitochondria, interacts with ferredoxin reductase (FDxR), which transfers electrons from NADPH to CYP450 via ferredoxin (FDX), increasing the intracellular superoxide production [44,45]. The redox enzyme, p66shc, regulates the oxidative stress acting a different level. This adaptor protein may induce ROS generation by: (i) Translocating into mitochondria, after PKC-dependent phosphorylation, to transfer electrons from reduced cytochrome C to oxygen [46,47], (ii) activating the Rac-1-dependent plasma membrane NADPH oxidase [48], and (iii) downregulating the expression of antioxidant enzymes, such as glutathione peroxidase-1 and manganese superoxide dismutase (Mn-SOD) [49,50]. The enzymes monoamine oxidase (MAO-A and MAO-B) are located in the OMM and expressed in various mammalian tissues catalyze the oxidation of biogenic amines accompanied by the release of H_2_O_2_ [51]. Mitochondrial aconitase is an enzyme positioned in the matrix, the enzyme contains an iron-sulfur cluster that can be oxidized by O_2_^•−^ or H_2_O_2_ generating •OH [52].

Uncoupling proteins (UCPs) are IMM proteins that belong to a family of mitochondrial transporters regulating the proton transport across the IMM. In particular, UCPs determine an inducible proton leak by causing mild-uncoupling events. During mild-uncoupling process, the mitochondrial membrane potential (ΔΨ) and the reduction events in the ETC diminished, thereby resulting in decreases in ROS production from the ETC [53].

UCPs exists in three forms: UCP 1-3. UCP1 is primary found in mitochondria from brown adipose tissue, where it regulates the thermogenesis in response to cold exposure and modulates the ROS production. Consistently, UCP1 knockdown mice are characterized by low levels of ΔΨ, reduced O_2_ consumption rate, and increased ROS production [54]. In addition, it has been recently demonstrated that UCP1 regulates the ROS production also in kidney. Deletion of UCP1 results in increased oxidative stress in kidney and exacerbates an ischemic condition or a nephrotoxic drug-induced acute kidney injury. Contrarily, viral-based expression of UCP1 suppresses the mtROS production and alleviates the induced kidney injury [55]. UCP2 and UCP3 mRNA expression are ubiquitous and their proteins are present in different tissues, in particular skeletal muscle, central nervous system, pancreas, and spleen. UCP2 and UCP3 protect mitochondrial proteins from endogenous ROS (such as O_2_^•−^), and UCP3 and UCP2 knockout models present increased ROS levels. In addition, it has been demonstrated that ROS, 4-hydroxy-2-nonenal (4-HNE), and lipid peroxidation determine activation of UCPs [56,57]. These findings suggest that UCPs exist in a feed-back mechanism, where increased ROS production activates a protective mechanism (uncoupling events) necessary to reduce the ROS formation and the consequent ROS damage. UCP2 has also an important role during insulin secretion in pancreatic β cells. In fact, UCP2 contributes to regulate the intracellular ROS levels of β cells, thereby controlling the excessive glucose-stimulated insulin secretion (GSIS) [58]. UCP2 represent an essential metabolite transporter that regulates the mitochondrial export into the cytosol of metabolites: malate, oxaloacetate and aspartate. Because of this transport, UCP2 prevents the oxidation of glucose and sustains the glutaminolysis [59]. Another protein that preserves an efficient electron transport and control the ROS production is hexokinase (HK) 2. HK2 is recruited to the OMM where it binds itself to the ADP/ATP exchange complex formed by VDAC and ANT. The role of HK2 is to provide continuously ADP in order to generate the ADP/ATP recycling mechanism essential to preserve the optimal respiration rate, thereby preventing the dangerous electron leak producing ROS [60]. In line with this evidence, HK2 knockdown and the dissociation of HK2 from mitochondria increase ROS production and activate the mitochondrial permeability transition (MPT) both in vivo and in vitro [61]. Accordingly, HK2 overexpression decreases the mtROS levels [61].

Sirtuins (SIRTs) are nicotinamide adenine dinucleotide (NAD)-dependent histone deacetylases that play pivotal role in diverse cellular functions, including protein acetylation and deacetylation, metabolism, mitochondrial functioning, and cell survival. In addition, all seven SIRTs isoforms have been associated to process related to antioxidant and redox signaling. In particular, the functions of SIRT1 (the most well-studied member) have been associated to mediate protection from ROS. At demonstration, small molecule activators of SIRT1, such as SRT1720, determine activation of SOD2 levels, accompanied by reduction in the formation of 4-HNE and in protein carbonylation levels [62]. SIRT1 can be also directly activated by oxidative stress and is required for DNA repairing mechanisms following H_2_O_2_-induced damage [63]. Similarly, the antioxidant compound resveratrol activates SIRT1 to protect cells from H_2_O_2_-induced cell death [64]. Another important SIRTs modulating ROS levels is SIRT3. Downregulation of SIRT3 is associated with increases of ROS production and activation of the protumorigenic transcription factor HIF-1α [65]. Furthermore, SIRT3 may activate the human 8-oxoguanine-DNA glycosylase 1 (OGG1), which is an enzyme crucial for the repairing of the mtDNA [66].

However, the levels of mtROS are conditioned by the specific action of mitochondrial antioxidant systems, recruited to detox the radicals produced. SODs are the antioxidant enzymes that convert O_2_^•−^ to H_2_O_2_ (Figure 1). Two isoforms control the level of O_2_^•−^: SOD1/copper-zinc superoxide dismutase (Cu, Zn-SOD) and SOD2/ Mn-SOD isoform. SOD1 is widely distributed throughout the cell, cytoplasm, nucleus, and intermembrane space of mitochondria (IMS), whereas SOD2 is expressed only in the mitochondrial matrix [67] (Figure 1). The rapid conversion into H_2_O_2_ is counteract by catalase and glutathione peroxidase (GPX), which neutralize H_2_O_2_ in H_2_O and oxygen [68,69] (Figure 1). Catalase is mainly located to cytosol, indicating that the scavenge capability in mitochondria it is leaved to GPX [70]. So far, in mammals, there have been eight GPX isoforms identified, where GPX1-4 and GPX6 are selenoproteins with a selenocysteine as catalytic moiety and only GPX1 and GPX4 are expressed to mitochondria. The GPX-dependent scavenge capability is associated to use glutathione (GSH) as cofactor and electron source to neutralize H_2_O_2_.

## 3. Mitochondrial Oxidative Stress and Mito-Inflammation in Inflammatory-Related Diseases

Mitochondrion is historically defined as energy powerhouse and arbiter of cellular destiny. Now, evidence assign to this organelle the supplementary role of “hub” in inflammation, becoming a druggable target to modulate the amplitude of inflammatory response and, eventually, its exacerbation. A new concept is arising around mitochondria and inflammation: The mito-inflammation, a mitochondria-related compartmentalized inflammatory response, where the organelle acts both downstream in intracellular signaling pathways triggered by exogenous pathogen-associated molecular patterns (PAMPs), and as font of mitochondrial damage-associated molecular patterns (mtDAMPs). During mito-inflammation, mtROS, mtDNA, ATP, cardiolipin, and mitochondrial Ca^2+^ are reversed in the cytosol or in the extracellular milieu to induce the expression and release of numerous pro-inflammatory mediators [71,72,73]. Among mtDAMPs, mtROS may directly act into the organelle, promoting oxidative damage to intra-organelle molecules and mtDNA, or freely move through the OMM (Figure 2). Once in the cytosol, mtROS may trigger the activation of pro-inflammatory signaling pathways, in particular, induce the activation of redox-sensitive transcription factors, such as nuclear factor kappa B (NF-kB), HIF and AP-1, which contribute to production of pro-inflammatory cytokines, including IL−1 and IL−8 (Figure 2) [74,75,76,77,78].

Oxidative damage to mtDNA and mitochondrial proteins alter the OXPHOS activity with several implications on ΔΨ and structure, producing in turn additional mtROS. Oxidized mtDNA is released from matrix mediating impaired mitophagy, Ca^2+^- and oxidative-dependent mitochondrial PTP opening, and OMM permeabilization [72,79]. In the cytosol, or in the extracellular milieu, mtROS and oxidized mtDNA may also be sensed by germline pattern recognition receptors (PPRs), localized at the plasma membrane and cytosol in immune and tissue resident cells [80]. These receptors are divided in four main sub-families on the basis of their location, function, and specific ligand: Membrane bound Toll-like receptors (TLRs), C-type lectin receptors (CLRs), the cytosolic NOD (nucleotide-binding oligomerization domain)-like receptors (NLRs), and RIG (retinoic acid- inducible gene)-l-like receptors (RLRs) [81]. 

In macrophages, binding of TLR-1, TLR-2, and TLR-4 induce the tumor necrosis factor receptor-associated factor 6 (TRAF-6) to move to mitochondria to induce antibacterial activity through mtROS [82]. It engages and ubiquitinates the ECSIT (evolutionarily conserved signaling intermediate in Toll pathways), which assembles the membrane arm complex I, promoting the translocation of mitochondria to the phagosomal membrane and the ECSIT-dependent macrophage oxidative burst useful to kill the engulfed microbes [82,83]

While TLR receptor have a plasma membrane localization, RLRs have a cytosol distribution. Among them, RIG-l-like (RIG-I) and melanoma differentiation associated gene 5 (MDA5) receptors can respectively sense short and long viral dsRNA. Once these proteins have been bound by cytosolic viral RNA, they interact with the mitochondrial antiviral signaling protein (MAVS) at the OMM. This coupling activates and promotes MAVS oligomerization leading to the activation of transcription factors IRF3, IRF7, and NF-kB, which induce the synthesis of interferon type-I and of antiviral molecules (Figure 2) [84]. MAVS interacts with NLR family member X1 (NLRX1), a regulator of mitochondrial antiviral immunity that localizes to OMM to block the MAVS-mediated interferon promoter and antiviral activity [85]. The strong connection between mtROS and MAVS has been enforced by evidences that linked an increase in mtROS production to an amplification of RLR-signaling and a mtROS-dependent modulation of the biophysical properties of the OMM required for MAVS oligomerization [86,87,88].

Another class of cytosolic PPRs activated by PAMPs and/or mtDAMPs are NLR proteins, where most of them after activation, form a multi-protein complex termed “inflammasome”, which leads to caspase-1 maturation and secretion of IL−1 and IL−18 [89]. A defined role for mitochondria in NLR activation and function has been confirmed for NLRX1, NLR family pyrin domain containing 3 (NLRP3), and NLR family CARD domain containing 4 (NLRC4/IPAF) [90]. The activation of the well-characterized inflammasome NLRP3 is split in two phases: Priming and activation. In the priming step, the expression and post-translational modifications of different inflammasome components (including NLRP3, NLRC4, pro-IL−1, pro-IL−18) are induced in response to pro-inflammatory stimuli and consequent NF-kB activation (Figure 2) [91]. The activation is controlled by a wide range of signals and requires a physical interaction with mitochondria for the assembly of NLRP3-inflammasome and the subsequent auto-cleavage of pro caspase-1, responsible for the production of mature cytokines IL−1 and IL−18 (Figure 2) [92]. Mitochondrial localization, due by MAVS and mitochondrial-anchored protein ligase (MAPL) binding, has a central role in the modulation of inflammasome response not only because of its role of scaffold, but also because this interaction favors the activation of NLRP3 through the release of mtROS and mtDAMPS (e.g., mtDNA) [93,94].

Moreover, it has been shown how the priming and the activation phases of NLRP3 inflammasome is linked to the new synthesis of mtDNA for inflammasome activation, highlighting the role of mitochondria in both the steps required [95]. In addition, NLRC4/IPAF inflammasome may be activated by oxidized mtDNA. This inflammasome is known to be activated by many pathogens, such as *Pseudomonas aeruginosa*, and their products by activation of TLRs on the plasma membrane via the microbial type III secretion system (T3SS) [96,97]. Nevertheless, few years ago an alternative mechanism of activation promoted by direct binding of oxidized mtDNA has been identified in bone-marrow macrophages [98].

The contribution of mtROS in inflammation is strictly influenced by mitochondrial status. mtROS levels increase especially during mitochondria malfunctioning, that cover a wide range of abnormalities from accumulation of unfolded proteins or excessive Ca^2+^, to OXPHOS impairment. Besides their role of signaling molecules at low concentrations and of inflammatory response activators at moderate concentrations, when mtROS concentration becomes too high, they are also responsible of cellular injuries. In order to avoid cell damage, the production of mtROS is fine-tuned by both mitochondrial antioxidant systems and mitochondrial stress responses that trigger the restore of mitochondrial homeostasis. Functional fusion complementation, mitophagy, and mitochondrial unfolded protein response (UPR^mt^) intervene to recover and preserve the mitochondrial homeostasis to regulate metabolism and innate immune response and cell viability. Mitochondrial fusion compensation optimizes the functional efficiency of organelle under stressful conditions, allowing the exchange of materials among partially damaged mitochondria [99]. Mitophagy neutralizes excess of mtROS, oxidized mtDNA, and other mtDAMPs relevant for inflammation, removing dysfunctional mitochondria mediating lysosomal degradation [100]. Mitochondrial-targeted kinase PINK1 and E3 ubiquitin ligase Parkin are the principal actors in the mitophagic response [101]. Parkin is recruited to OMM by altered mitochondrial import of PINK1 in depolarized mitochondria, where catalyzes the ubiquitination of OMM proteins to sequester the organelle in autophagosome, while PINK1 contributes to strengthen the mitophagy, phosphorylating both parkin and ubiquitin; and recruiting the mitophagic receptors NDP-52 and optineurin to mitochondria [102,103]. Finally, the mitochondrial unfolded protein response (UPR^mt^) is a transcriptional response activated by dysfunctional mitochondria, where upon mitochondrial stress, Activated Transcription Factor 5 (ATF5) fails to be imported into mitochondria and moves to nucleus to induce the transcription of mitochondrial chaperones and proteases, ROS detoxification protein, and innate immune genes [104,105,106]. UPR^mt^ induces also the synthesis and secretion of nuclear-encoding and mitochondrial derived mediators, called mitochondrial cytokines or mitokines, able to reshape cell metabolism and viability [98,107,108]. Although, the signaling role of these mitokines has not yet been fully established, they may be considered pivotal actors for the modulation of systemic response, such as metabolism and or inflammation, during a disease progression. Furthermore, the excess of mitochondrial Ca^2+^ levels is critical for oxidative stress and inflammation. Perturbation of mitochondrial Ca^2+^ signaling boosts the mtROS production with consequent repercussions on the mitochondrial stress responses, inflammasomes activations and release of proinflammatory mediators [109]. The mtROS production correlates with the mitochondrial metabolic rate, which in turn, determines the effects of mitochondrial Ca^2+^ signaling on mtROS levels [110]. Mitochondrial Ca^2+^ signaling may promote mtROS production: (i) directly, by stimulating mitochondrial resident ROS-generating enzymes, such as -ketoglutarate and glycerol 3-phosphate dehydrogenase [111,112]; (ii) indirectly, by the Ca^2+^-dependent activation of nitric oxide synthase, which mediating nitric oxide blocks the mitochondrial complex IV; and (iii) or via reverse electron transport induced by Ca^2+^-dependent mitochondrial membrane depolarization [113,114]. In turn, mtROS contribute to perturb the Ca^2+^ signaling affecting the activity of receptors, Ca^2+^-effectors, and molecules involved in Ca^2+^ signaling pathways, reshaping intracellular, and compartmentalized Ca^2+^ signals [110]. An example is given by mitochondrial Ca^2+^ uniporter (MCU), which post-translational modification on cysteine at position 97, mediated by oxidative stress, induces clustering and persistent channel activation, leading to increased mitochondrial Ca^2+^-uptake [115].

The loss of mitochondrial homeostasis and the excessive accumulation of mtROS have been demonstrated to be linked to development of various pathologies, such cancer, diabetes, neurodegeneration, and cardiovascular diseases. This review is aimed at discussing some pathological conditions associated with an inflammatory state, which is strictly conditioned by the abnormal presence of these two factors, where mitochondrial dysfunction and mtROS become the principal actors in these pathological sceneries.

### 3.1. Cancer

The mtROS are involved in the different phases of tumorigenesis. In the initiation and promotion stage, mtROS induce mtDNA oxidation and damage, promoting mutations and structural alterations to mtDNA with consequent effects on gene expression and mitochondrial signaling. All these conditions, in turn, favor the cell proliferation and promote the apoptotic evasion in the progression stage. Wang et al., examining human lung, bladder and head and neck cancers, showed that the mutation in mtDNA were about 20-200 times more frequent respect to nuclear DNA [116]. The mtROS are responsible not only for the genetic instability but also for influencing the inflammatory microenvironments of tumors and to induce the activation of oxidative-sensitive transcription factors, such as HIF, which modulate the energy status in cancer cells [117,118]. Cancer cells adopt a different metabolism to produce energy, where the use of aerobic glycolysis results is preferred with respect to mitochondrial oxidative phosphorylation. This metabolic switch is known as the “Warburg effect” and is characterized by the fact that cancer cells use glucose and excrete lactate. This phenomenon was unveiled in the 1920s by Warburg and Cori. Subsequently, they also observed that mitochondria of cancer cells were dysfunctional. Several other investigations confirmed the Warburg effect throughout the years. Fantin et al. in 2006 demonstrated that it was possible to prevent the tumor progression by blocking the conversion of pyruvate into lactate by inhibiting lactate dehydrogenase [119]. In the same year, another work demonstrated that by activating the mitochondrial metabolism, in particular OXPHOS, the cancer growth arrested [120]. Therefore, it is well assumed that cancer cells avidly consume glucose for their maintenance. Consistent with this, the Warburg effect has been observed in many cancer types. In colorectal carcinoma, a strong correlation was observed between the malignant potential of the tumor and the levels of the expression of the glucose transporter (GLUT) proteins, responsible for the cellular glucose uptake [121]. Increased levels of GLUT have been also evidenced in human breast cancer [122]. Another example may be found in lung cancer, where a great dependency on glycolysis and a parallel impairment in mitochondrial respiration was found [123]. Finally, it has been demonstrated that aerobic glycolysis may be not only a consequence of carcinogenesis, but also favors the transformation process [124].

However, last decades have been characterized by very great improvements in the field of the scientific research. These advances have permitted a deeper and more detailed analysis of the continuous metabolic changes that happen in cancer cells, and in particular, it has been demonstrated that the Warburg effect is not consistent in all cancer types. In fact, in a range of tumor types, the mitochondria remain functional [125,126]. This happens in ovarian cancer, where it has not only been found to have a high dependence on mitochondria of cancer cells, but also that anti proliferative agents reduce the tumor growth by suppressing the mitochondrial metabolism [127]. By analyzing the metabolism in non-small cell lung cancer (NSCLC) patients in vivo, a great metabolic heterogeneity has been observed between different tumor regions: Some regions were characterized by high glycolytic rates, while others presented elevated levels of complete glucose oxidation [128]. High mitochondrial functioning has been found in liver cancers and in metastatic brain tumors [125,126,129]. Overall, these findings suggest that during the tumorigenesis, cancer cells may undergo a metabolic reprogramming, in which the Warburg effect represents a key event. Although, changes in glucose metabolism during tumor initiation and growth affect cellular processes that may generate ROS [130]. 

Indeed, oncogenes and tumor suppressor genes in cancer cells promote mtROS production, including oncogenic H-Ras that induces mtROS formation for mitogenic signaling [131,132]. This increment in ROS levels is due to mitochondrial hyperactivity mediated by major changes in Ψ and Ca^2+^-affinity/accumulation. The oncogenic mitochondrial hyperactivity is not sustainable for all the stages of tumor process. mtROS accumulation may damage the organelle, exacerbating the mitochondrial oncogenic stress. Under these conditions, the cancer cells active the mitochondrial stress responses, such as mitophagy and UPR^mt^, to restore the mitochondrial integrity potentiating the cellular survivor and resistance to stress [133]. mtROS produced by cancer cells are signaling molecules released in tumor microenvironment to condition cancer-associated cells and tumor-infiltrating leukocytes [117]. mtROS affect the function of cancer-associated fibroblasts, inducing differentiation and metabolic reprogramming to sustain and augment the tumorigenesis [134,135,136]. Although T-cells use complex I and III of ETC to generate a low level of mtROS to induce T-cell activation, the excessive accumulation of ROS in the tumor microenvironment has an immunosuppressive effect on the tumor-infiltrating immune cells [134]. High levels of ROS block the proliferation of infiltrating T-cells and inhibit their anti-tumor function [137]. These effects are counteracted with mtROS scavengers, such as MitoQ and MitoTEMPO, which restore the T-cell activation [138]. The controversial role of ROS in tumor microenvironment is represented by the fact that ROS are also produced by tumor-associated macrophage and myeloid-derived suppressor cells. In the case of macrophages, the ROS are implicated in cell activation and in killing processes, while for the myeloid-derived suppressor cells, the ROS are implicated as immunosuppressive signals to regulate immune cell functions [139,140].

### 3.2. Pulmonary Diseases

The mtROS formation is excessive in Cystic Fibrosis (CF) cells [73]. This pathology is characterized by genetic defects of cystic fibrosis transmembrane conductance regulator (CFTR) gene and recurrent pulmonary infection, which cause chronic inflammation of airways and respiratory failure. The persistent bacterial infections, in particular of *P. aeruginosa*, induce mitochondrial Ca^2+^-overload and mtROS formation in human CF bronchial cells, which in turn promoted the activation of NLRP3 and NLRC4 inflammasome and consequent release of IL−1 and IL−18 [109]. This interplay between NLRP3 and NLRC4 inflammasome has also been observed in CFTR-null mice, in alveolar CF macrophage, and in CF neutrophils, due to mtROS and direct binding with oxidized mtDNA [98,141]. Limiting the abnormal mitochondrial Ca^2+^-uptake in CF and mediating pharmacological inhibition of MCU, with KB-R7943, attenuated the pathogen-dependent ROS production and mitochondrial dysfunction, preventing the activation of NLRP3 inflammasome and UPR^mt^ in vitro and in vivo [142].

The high levels of mtROS promote additional mitochondrial impairments in a feed-back stimulatory manner that sustains the activation of oxidative-sensitive transcription factors, which exacerbate the chronic pulmonary inflammation [2,143]. In fact, NF-kB, HIF-1, and AP-1 are hyperactivated in CF, favoring an elevated production of cytokines and chemokines, such as IL−8, and priming the inflammasome NLRP3 and its members. Their activation is, due to intrinsic defects, associated with defective CFTR, such as the oxidative stress and abnormal intracellular Ca^2+^ signaling [144,145,146,147,148,149].

The oxidative-sensitive nuclear recruitment of NF-kB also has a pivotal role in the chronic obstructive pulmonary disease (COPD). Here, its involvement is a determinant in sustaining the chronic inflammatory response through the up-regulation of different pro-inflammatory and chemotactic genes, including IL−8 and TNF-α, both important to COPD pathogenesis [150].

### 3.3. Gastrointestinal Disorders

In the gastrointestinal (GI) tract the NOX and xanthine oxidase (XO) systems represent the primary ROS producers. Notably, XO, which is mainly expressed in the liver and in the intestinal mucosa, catalyzes the oxidation of hypoxanthine (HX) to xanthine and the conversion of xanthine to uric acid. During these two reactions O_2_^·−^ is produced. Excessive ROS production in GI is linked to inflammation and may be the cause of severe cellular damages that can disrupt the tract barrier of GI, thus determining gut permeability and contributing to different GI disorders. Gastroesophageal reflux (GR) represents a very common disorder, and all elements associated with GR, such as bile salts and acids, have been found to be potent ROS inducers as well as a primary cause of loss of antioxidant defenses. As a result, the squamous epithelium of esophagus undergoes erosion and ulceration [151]. Accordingly, by injecting antioxidants in a rat duodenogastroesophageal reflux model, ROS amounts and esophagitis resulted reduced [152]. Gastritis represents the inflammation of the stomach mucosa. When gastritis is chronic, it may be a cause of peptic ulcer disease. Different conditions provoke gastritis, such as bacterial infection, stress, cigarette smoke, and excessive alcohol consumption. All of them contribute to accumulating ROS and activate inflammation, with a consequent infiltration of neutrophils and macrophages in the gastric mucosa that can exacerbate the oxidative stress [153]. Interestingly, it has been demonstrated that RNS results in being protective for gastric tract. Indeed, NO radicals stimulate mucus secretion and inhibit the expression of adhesion molecule in the gastric epithelium. In this manner, ROS-mediated ulceration is prevented as neutrophils cannot adhere to the gastric mucosa [154]. In human intestinal diseases, the intracellular ROS levels are increased, generating cell stress and a reduction in the diversity of microbial community in the gut [155]. An increase of 10-100 times in mucosal ROS concentration has been observed in ulcerative colitis, in gastroduodenal mucosal inflammation, Chron’s, and inflammatory bowel diseases [156,157,158]. The epithelium barrier works to protect itself and the gastrointestinal district by stressors, such as pathogens, secreting mucus and antimicrobial peptides and low levels of ROS [159]. In this, the mitochondrial signaling and concomitant metabolic changes contribute significantly to intestinal equilibrium. In bowel epithelium, the mitochondrial impairment induces a metabolic imbalance that causes reduction of stemness and generation of dysfunctional Paneth cells, which predicts the Chron’s disease recurrence [160]. Indeed, an excessive increase in ROS production in gut has detrimental effects on the gastrointestinal membranes with consequent repercussion on tissue permeability and microbial biodiversity. Many inflammatory-related gastrointestinal disorders are associated with dysbiosis, an imbalance of gut microbiome that is linked to the release of PAMPs that induce inflammation [161].

### 3.4. Cardiovascular Diseases

The importance of oxidative stress in cardiovascular diseases (CVDs) has been continuously demonstrated. Overproduction of ROS and other oxidative stress-related factors are indeed the primary cause of diverse CVDs, in particular during atherosclerosis and myocardiac infarction. Low-density lipoproteins (LDL) are major cholesterol carriers, and their levels play a crucial role in atherosclerosis. Increased oxidative stress determines conversion of LDL in oxidized LDL (ox-LDL), which represent pathogenic, immunogenic, and atherogenic particles. In this conformation, LDL can enter the monocyte-macrophage system present in the arterial wall and cause the atherosclerotic process [162,163]. In particular, ox-LDL may increase the expression of the intercellular adhesion molecule-1 (ICAM-1) and vascular-cell adhesion molecule-1 (VCAM-1), improving the adhesion of monocytes to the arterial endothelium [164]. Ox-LDL also determine the secretion of monocyte chemotactic protein-1 (MCP-1) and monocyte colony stimulating factor (mCSF) by stimulating smooth muscle cells (SMCs) and endothelial cells (ECs) migration and proliferation [165]. Increase in inflammatory levels during atherosclerosis may also be mediated by oxidative stress. High ROS levels, in particular H_2_O_2_, directly stimulate macrophages to express chemokines and inflammatory cytokines to boost the inflammatory response at the site of the atherosclerotic endothelial injury. The immune receptor NLRP3 is also involved in atherosclerosis. Consistently, NLRP3 components are present in SMCs and ECs [166]. Most importantly, high NLRP3 levels characterize patients with coronary atherosclerosis and correlate with the severity of disease and with concomitant. Interestingly, increased NLRP3 levels also correlate with the presence of concomitant risk factors in patients with coronary artery disease. In particular, it has demonstrated that the presence of oxLDL primes and activates NLRP3 components. Accordingly, by treating endothelial cells with an inhibitor of component of NLRP3 ASK1 (GS-4997) it is possible to attenuate the inflammatory process, the ROS production, and, most importantly, the effects of oxLDL on the cholesterol efflux [167]. Cigarette smoking represent one of the major risk factors for the development of atherosclerotic plaque. It has been demonstrated that nicotine improves the mtROS levels and activate a molecular pathway where NLRP3 and pyroptosis cooperate to promote monocyte recruitment, release of pro-inflammatory factors, and pyroptotic cell death of ECs [168]. In particular, OGG1 is responsible for removing 7,8-dihydro-8-oxo-2′-deoxyguanosine (8-OH-dG), the most abundant form of oxidative DNA damage [169]. It has been demonstrated that OGG1 downregulation leads to increased mtDNA damage as well as activation of NLRP3 inflammasome in atherosclerotic lesion [170]. Consistent with this, increased mtDNA damage levels have been observed in human atherosclerotic plaques (Yu et al., 2013).

Ischemic cardiac injury happens during myocardial infarction and represents the result of an occlusion of a coronary vessel, in which cardiac cells suffer a hypoxic condition, ATP depletion, and mitochondrial impairments. It has been demonstrated that during the ischemic condition complexes I and III result compromised, favoring the excessive mtROS production. This situation provokes severe damage to different phospholipid components of mitochondrial membranes, such as cardiolipin, leading to impairments and destabilization of ETC members, depletion of ATP production, mitochondrial permeability transition, and finally, cell death. Accordingly, to these findings, for several years, a quick restore of the blood supply (reperfusion) was though essential to limit the cellular damage. Consistently, reperfusion restores oxygen delivery, ATP production, and mitochondrial Ca^2+^ levels. At the same time, the reintroduction of blood rich in oxygen is deleterious for hypoxic tissue, which triggers an uncontrolled mtROS production by complexes I and III, determining mPTP opening, mitochondrial membranes permeabilization, and cell death [171,172,173]. Recently, it has been proposed that during ischemia/reperfusion (IR), the ETC may be not the only source of mtROS, indicating that other mtROS producers are involved [174].

As reported above, MAO are proteins localized on the OMM and are responsible for the generation of H_2_O_2_ [175]. It has been demonstrated that during IR the MAO-A expression increases in cardiomyocyte and it is responsible for producing excessive H_2_O_2_ production, which provokes mitochondrial damage. Interestingly, it has been proposed that during IR, the excessive ROS production mediated by MAO-A is sufficient to block the activity of sphingosine kinase, degrades sphingosine-1-phosphate (S1P) and increases the ceramide levels [176]. Notably, S1P suppresses the ceramide-mediated cell death [177]. Consistent with these findings, inhibition of MAO-A reduces mitochondrial lipid peroxidation and limits the cardiac infarct size, representing a potential therapeutic intervention against cardiac IR [178].

Another important contributor for mtROS production is the protein p66Shc, which is expressed in cardiomyocyte and is responsible for regulating different cellular processes [46,179,180]. Genetic silencing of p66Shc reduces the mtROS production and limits the lipid peroxidation, determining cardioprotective effects during IR model.

Among the different mechanisms through which ROS provoke IR injury of the heart, ROS-mediated inflammasome activation has an important contributor. In fact, mediator of inflammasomes, such as IL−1 and ASC, have key roles during cardiac IR injury and their inhibition exerts protective role against myocardium in mice exposed to IR [181,182]. In addition, it has been suggested that the role of NLRP3 in cardiac IR may be related to the member of α-arrestin protein superfamily, Thioredoxin-interacting protein (TXNIP). In a recent work, it has been demonstrated that during myocardial IR the interaction between TXNIP and NLRP3 resulted in an increase, and that following intramyocardial injection of TXNIP siRNA, the NLRP3 activation as well as the infarct size was reduced [183]. Moreover, when mtROS scavengers were added, the association between TXNIP and NLRP3 and the inflammasome activation were abrogated, suggesting that mtROS are responsible for activating the TXNIP -mediated activation of NLRP3 [183].

### 3.5. Neurodegenerative Disorders

Alzheimer’s (AD), Parkinson’s (PD), and Huntington Disease (HD) are neurodegenerative disorders with significant motor and cognitive decline, and, to date, incurable diseases associated to aging [184].

The progression of neurodegenerative diseases is associated with neuroinflammation promoted by protein aggregation and/or neuronal damage via damage-associated molecular patterns (DAMPs) and consequent activation of PRRs, such as CR3 and TLR-4, with the pivotal contribution of mtROS [185,186]. The activation of these receptors induces neuroinflammation mediating the recruitment pro-inflammatory signaling transducers, such as NLRP3 and NF-kB, which, activated by oxidative stress, promote the synthesis and release of pro-inflammatory mediators [187,188,189]. The susceptibility of neural tissue to oxidative stress is not only due to elevated ROS production but also reduced regeneration capacity of neurons and their modest antioxidant defenses [190]. The immune responses in neurodegenerative diseases are regulated by microglia cells, which mediate both protective and deleterious responses [191].

In AD, the release of pro-inflammatory cytokines, IL−1, is mediated by assembled NLRP3 inflammasome in activated microglia in response to aggregation of amyloid- proteins and consequent plaque formation [192,193,194]. Indeed, amyloid- has been shown to target the mitochondria, affecting the mitochondrial enzymes, alcohol dehydrogenase and cytochrome C oxidase [195]. A direct link between mtROS and the pathology has been observed also in transgenic mouse model and in human brain tissue [196,197]. The neurotoxicity in AD is also favored by activation of NLRP3 inflammasome, which induces tau abnormality favoring tau phosphorylation and aggregation [198,199]. Its deactivation has protective effects in AD [200]. Mitochondrial dysfunction, mtROS formation, and consequent NLRP3 inflammasome activation are also associated with bipolar and intracerebral hemorrhage disorders [201,202].

The progression of pathology is also conditioned by insidious aspect of mitochondrial signaling under oxidative stress, such as the persistent activation of UPR^mt^, which may exacerbate the disruption of neurons in vivo or induce the release of neuroprotective mitokines in AD, such as humanin [202,203]. Humanin reduce the neuroinflammation and the oxidative stress-induced injury in AD, diminishing the level of protein aggregation and of plaque deposition [204,205]. 

The production of ROS and the activation of NLRP3 is sustained and elevated also in PD, where microglia cells, in response to Lewy bodies and -synuclein protein, also release cytokines IL−6, TNF, and prostaglandins [206,207,208]. In particular, the alterations in complex I of ETC are the primary source of mtROS formation in PD patients, causing nigrostriatal degeneration observed in PD [209,210,211].

In HD patients, it is the activity of complex II of ETC that is diminished [212], with significant repercussions on mitochondria and mtDNA integrity, the cause of massive inflammation that damages the striatum [213]. In this area, only the pro-inflammatory cytokines IL−1 and TNF were increased, while IL−6, IL−8, and MMP-9 were up-regulated in cortex and cerebellum, two districts that normally are spared in HD [214].

The direct involvement of mitochondria in AD and PD is confirmed by the compartmentalized ROS suppression mediating mitoTEMPO, where the treatment reduced the expression of pro-inflammatory mediators, IL−1, IL−6, TNF, iNOS, and COX-2 in murine microglia cells, limiting the nuclear translocation of NF-kB and MAPKs [215].

### 3.6. Diabetes

Diabetes is described as a multifactorial and complex metabolic syndrome characterized by deficit in metabolism of carbohydrates, fats, and proteins. Consistent with this, hyperglycemia represents the main pathological condition.

A hyperglycemic condition can determine increase the mitochondrial flux determining increase in oxidative stress [216]. This elevation may be due to an increase in production of ROS/RNS (such as ONOO−, OH, 8-OHdG, and H_2_O_2_) through the ETC as well as decreases of antioxidant defense systems, in particular, catalase (CAT), glutathione peroxidase (GSH), and SOD [217,218].

In addition to this, nutrient overload determines increases in insulin synthesis demand, which forces -cells to product insulin [219]. ER is the primary organelle involved in protein synthesis. An uncontrolled insulin demand promotes an increase in disulfide bond formation for correct protein folding at ER, a condition that increases the ROS formation [220]. As a consequence, protein folding fails and ER functions are impaired [221]. Mitochondrial oxidative stress and ER stress create a vicious cycle that increases oxidative stress and affects further the functioning of these determinant organelle. Considering that both ER and mitochondria are crucial for controlling the glucose levels in blood and in -cells, the hyperglycemic condition becomes chronic. When this situation occurs, it is highly harmful for both insulin secretion and survival of -cells and diabetes may develop.

In addition, the excess in ROS production may deregulate the expression of important key factors (such as MafA and PDX-1) necessary for the activity of different genes involved in insulin generation [222].

ROS-dependent hyperglycemic damage is also associated with other cellular processes, in particular inflammation, and one of the primary targets of hyperglycemic damage are the vascular endothelial cells (VES). Notably, VES are not able to regulate the intracellular glucose concentration and when blood presents excessive amounts of glucose, they cannot prevent the glucose entry. This condition, together with uncontrolled ROS levels, promote damage and vascular complications [223,224]. Consistently, it has been demonstrated that in human aortic endothelial cells, the inhibition of ROS production by uncoupling of ETC reduced the chemokine IL−8 expression induced by high level of glucose [225]. The hyperglycemia-induced monocyte-endothelial adhesion and successiv transmigration was also enhanced in human coronary artery, where the mtROS promoted the redox-sensitive NF-kB activation and the expression of adhesion molecules, such as ICAM-1 and VCAM-1 [226].

The activation of the nuclear transcription factor NF-kB in diabetes is the result of ROS generation promoted by the excessive glucose and by saturated fatty-acids. TLR-4 senses the excess of saturated fatty-acids favoring the oxidative-dependent up-regulation of inflammatory genes, which determine the markedly release of pro-inflammatory mediators involved in monocyte adhesion and chemotaxis, such as IL−8 [227,228]. 

Vascular tissue is not the only target for ROS-driven inflammation in diabetes. The presence of inflammatory partner has also been identified in pancreas. Indeed, activated macrophages are present in the pancreas of diabetic persons and are responsible for the damage and cell death of pancreatic islet through ROS [229]. In this context, it has been proved that by using ROS scavenger, it is possible to reduce the inflammatory events and increase the pancreatic cell survival [229].

## 4. Conclusions

In recent years, the mitochondria have been increasingly assuming a key role in the pathogenesis of human diseases. Always considered the cellular powerhouse, the mitochondrion also acts as a strategic hub in diverse cellular process, such as inflammation. The mitochondrion is involved in the regulation of inflammation through multiple mechanisms, not only by releasing mDAMPs but also by affecting the mitochondrial stress responses or influencing the inter-organelle communications between ER and/or nucleus. Their involvement in the inflammation has suggested a new concept, the mito-inflammation, a compartmentalized inflammatory response that may be targeted to cure a wide range of inflammatory-associated pathogenic conditions. Consistently, different studies show how mtROS are the principal actors, fuel that feeds and sustains this process. It has been established that mtROS are endogenously signaling molecules, involved in a complexity of interactions. At low levels, they are necessary to regulate cellular functions and stimulate the mitohormesis, while their excessive accumulation is damaging to cells. Understanding the mechanisms by which mtROS communicate will aid in the development of mitochondrial targeted therapies for human inflammatory-related diseases. The mitochondrial antioxidant is the compartmentalized antioxidant strategy finer to counteract the ROS production to mitochondria. To this, targeted antioxidants have been developed, such as MitoQ and SkQ1 that reduce the severity of many inflammatory-related pathologies [230]. The mitochondrially antioxidant strategy has demonstrated cardioprotective efficacy decreasing the mtROS accumulation in many cardiac diseases [231]. MitoTEMPO and MitoQ protect from reperfusion injury and heart failure [232,233,234,235], while the mitochondrial Szeto-Schiller-31 peptide prevents oxidative stress and mitochondrial dysfunction targeting cardiolipin [236]. mtROS formation may be inhibited using MAO inhibitors. Several MAO inhibitors are clinically available for the treatment of neurological disorders and are currently the better compartmentalized strategy to counteract the mtROS formation in clinical use [237].

Many questions regarding the molecular function of mtROS and of mito-inflammation remain to be addressed. These findings and those that will be obtained in the coming years will increasingly reinforce the contribution of mitochondria and the mitochondrial quality control machinery in the mito-inflammation regulation, warranting the efforts of researchers to develop new mitochondria-targeted therapies.

## Data Availability

Not applicable.
